# PredicT-ML: a tool for automating machine learning model building with big clinical data

**DOI:** 10.1186/s13755-016-0018-1

**Published:** 2016-06-08

**Authors:** Gang Luo

**Affiliations:** Department of Biomedical Informatics, University of Utah, Suite 140, 421 Wakara Way, Salt Lake City, UT 84108 USA

**Keywords:** Machine learning, Automatic algorithm selection, Automatic hyper-parameter value selection, Automated temporal aggregation, Big clinical data

## Abstract

**Background:**

Predictive modeling is fundamental to transforming large clinical data sets, or “big clinical data,” into actionable knowledge for various healthcare applications. Machine learning is a major predictive modeling approach, but two barriers make its use in healthcare challenging. First, a machine learning tool user must choose an algorithm and assign one or more model parameters called hyper-parameters before model training. The algorithm and hyper-parameter values used typically impact model accuracy by over 40 %, but their selection requires many labor-intensive manual iterations that can be difficult even for computer scientists. Second, many clinical attributes are repeatedly recorded over time, requiring temporal aggregation before predictive modeling can be performed. Many labor-intensive manual iterations are required to identify a good pair of aggregation period and operator for each clinical attribute. Both barriers result in time and human resource bottlenecks, and preclude healthcare administrators and researchers from asking a series of what-if questions when probing opportunities to use predictive models to improve outcomes and reduce costs.

**Methods:**

This paper describes our design of and vision for PredicT-ML (prediction tool using machine learning), a software system that aims to overcome these barriers and automate machine learning model building with big clinical data.

**Results:**

The paper presents the detailed design of PredicT-ML.

**Conclusions:**

PredicT-ML will open the use of big clinical data to thousands of healthcare administrators and researchers and increase the ability to advance clinical research and improve healthcare.

## Background

### Challenges in building machine learning predictive models with big clinical data

Predictive modeling is vital to transforming large clinical data sets, or “big clinical data,” into actionable knowledge for various healthcare applications. Predictive models can guide clinical decision making and personalized medicine. For example, by determining an asthma patient’s risk of hospitalization within the next year, we can enroll patients at high risk in an asthma case management program [1].

As a major approach to predictive modeling, machine learning studies computer algorithms that improve automatically through experience, such as decision tree, random forest, neural network, and support vector machine [2]. A recent survey showed that 15 % of hospitals use machine learning predictive models for clinical purposes, and many more are considering it [3]. Historically, most machine learning algorithms were criticized for providing no explanation for their prediction results. Recently, a new method was developed to automatically explain prediction results of any machine learning model without losing accuracy [1, 4]. However, machine learning still presents two major challenges to use in healthcare, both badly supported by existing software such as Weka [5], KNIME, R, and RapidMiner [6].

### Challenge 1: efficiently and automatically selecting algorithms and hyper-parameter values

Every machine learning algorithm has two kinds of model parameters (see Table 1): ordinary parameters automatically learned or optimized in model training, and hyper-parameters manually set by the user of a machine learning tool before model training. Given a modeling problem like predicting whether an adult will develop type 2 diabetes, an analyst builds a model manually and iteratively. First, the analyst selects an algorithm from many applicable algorithms like the 39 classification algorithms in Weka [5]. Second, the analyst sets the chosen algorithm’s hyper-parameter values. Third, the analyst trains the model to automatically optimize the chosen algorithm’s ordinary parameters. If model accuracy is insufficient, the analyst changes hyper-parameter values and/or algorithm and re-builds the model. This process is iterated until the analyst obtains a model with sufficient accuracy, no longer has time, or cannot improve model accuracy. Numerous combinations of algorithms and hyper-parameter values result in hundreds or thousands of labor-intensive manual iterations to build a model, which can be difficult even for experts in machine learning [7].

**Table 1:** Some example machine learning (ML) algorithms, ordinary parameters and hyper-parameters

ML algorithm	Example ordinary parameters	Example hyper-parameters
Random forest	Input variable and threshold value selected at every internal node of a decision tree	Number of decision trees, number of input variables to evaluate at every internal node of a decision tree
Support vector machine	Support vectors, lagrange multiplier for every support vector	Kernel to use, degree of a polynomial kernel, *ε* for round-off error, regularization constant *C*, tolerance parameter

The algorithm and hyper-parameter values used affect model accuracy. Thornton et al. [7] showed for the 39 algorithms in Weka, the effect on model accuracy is on average 46 % on 21 data sets and 94 % on one data set. Even if considering only a few common algorithms such as support vector machine and random forest, the effect is still >20 % on 14 of 21 data sets. Moreover, the effective combination of an algorithm and hyper-parameter values varies by modeling problem. Researchers have explored automatic search for algorithms and hyper-parameter values [8]. Rising evidence shows that automatic search methods can obtain equivalent or better results than machine learning experts’ careful manual tuning [9, 10]. However, when many algorithms are considered, previous efforts such as MLbase [11, 12], hyperopt-sklearn [10], and Auto-WEKA [7] cannot quickly select good algorithms and hyper-parameter values on a large data set and have limited usefulness.

A key barrier to automatic search is the long time required to evaluate a combination of an algorithm and hyper-parameter values on the full data set. For instance, for the champion ensemble model of the Practice Fusion diabetes classification competition [13], it takes 2 days on a modern computer to train the model once on 9948 patients with 133 features, a.k.a. input or independent variables. To identify an effective combination, prior automation efforts evaluate numerous combinations on the full data set. Even when aborting long-running tests, ignoring ensembles of >5 base models, and allowing rather incomplete search that all impact search result quality, search time can be several days on a data set containing several thousand rows (data instances) and several dozen attributes [7]. Practically, search time can be thousands of times longer for four reasons: (1) Machine learning is an iterative process. If a set of clinical parameters generates low prediction accuracy, the analyst can add other clinical parameters that may be predictive. Every iteration needs a new search for algorithms and hyper-parameter values. (2) Ensembles of many base models often achieve higher accuracy. An ensemble model’s training time grows linearly with the number of base models. (3) A data set can contain many rows, e.g., from several healthcare systems. (4) A data set can have many attributes, e.g., obtained from genomic and/or textual data. The execution time of an algorithm frequently increases superlinearly with the number of rows and at least linearly with the number of attributes. A long search time precludes healthcare administrators and researchers from asking a series of what-if questions when probing opportunities to use predictive models to improve outcomes and reduce costs. An example question is: can we predict asthma readmission accurately enough to apply an intervention effectively? Moreover, personalized medicine requires solving many predictive modeling problems for various diseases and outcomes, where search time is a bottleneck even with a computer cluster. Regardless of whether search is conducted automatically or manually, a slow search speed often forces the search process to be stopped prematurely, leading to suboptimal model accuracy and outcomes of using models.

### Challenge 2: efficiently automating temporal aggregation of clinical attributes

Many clinical attributes are repeatedly recorded over time, requiring temporal aggregation before machine learning. For example, weight at each visit is aggregated to obtain a patient’s average weight over the past year. For each attribute, computing a temporal aggregate value requires specifying a pair of aggregation period and operator (e.g., average, maximum, count). Usually, healthcare professionals help specify pairs and computer scientists conduct computation. Many pairs can be clinically meaningful. Those producing high accuracy vary by predictive modeling problem and are often unknown beforehand.

Given a predictive modeling problem, an analyst manually identifies one or more temporal aggregation periods and operators for every clinical attribute, then builds the model. If model accuracy is insufficient, the analyst changes aggregation periods and operators for some attributes and re-builds the model. This labor-intensive process requires interactions between healthcare professionals and computer scientists and is often repeated hundreds of times, causing another time and human resource bottleneck, particularly when healthcare professionals have limited time for meeting and discussion. Also, no complete list of aggregation operators is available, requiring caution to not miss effective operators.

### Our new software

New methods are needed to efficiently automate building machine learning predictive models with big clinical data. To fill the gap, in this paper we describe the design of new software called PredicT-ML (prediction tool using machine learning) to automate the process. Conceptually, PredicT-ML is an automated version of Weka [5] for big clinical data, with added support for automated temporal aggregation. Computer scientists with advanced machine learning skills are scarce in healthcare institutes. PredicT-ML can be used by computer scientists when they work with healthcare administrators and researchers, can save effort for both healthcare professionals and computer scientists, reduce computing time, enable fast turnaround, lower the machine learning skill required of computer scientists, and increase their capacity of serving healthcare administrators’ and researchers’ predictive modeling needs. Compared to the current manual approach, PredicT-ML performs more tests systematically and can produce models achieving accuracy closer to the theoretical limit.

PredicT-ML provides an intuitive graphical user interface. The user can optionally specify feature selection techniques and machine learning algorithms to be explored, and/or some features that must be included in the model. PredicT-ML will efficiently and automatically select algorithms, feature selection techniques, hyper-parameter values, and temporal aggregation periods and operators. In real time, PredicT-ML displays projected model accuracy and estimated patient outcomes of using models as a function of time allotted for model building. If projections are unpromising, users can abort the automatic selection process, add more clinical parameters, and restart. Or, users can consider other ways of using predictive models to improve outcomes and reduce costs, e.g., by addressing another disease or prediction target. This enables users to rapidly ask a series of what-if questions when probing opportunities for improving healthcare, as even with our efficient techniques, automatic selection on a large data set can still take a relatively long time (e.g., a few hours).

PredicT-ML can run on a computer cluster for scalable parallel processing and perform fast iterative machine learning. For instance, an electronic medical record includes hundreds of thousands of clinical parameters [14, p. 56]. An analyst usually begins the analytical process with a few clinical parameters, then uses PredicT-ML to iteratively add more clinical parameters for analysis until reaching sufficient prediction accuracy. PredicT-ML can export temporally aggregated data in standard file formats such as comma-separated values (CSV) for use by other software systems, making PredicT-ML foundationally and structurally interoperable [15]. Internally, PredicT-ML keeps data in relational table formats accepted by many software packages. This will facilitate adding new functions such as visualization, making PredicT-ML extensible.

PredicT-ML is generalizable to various diseases. PredicT-ML’s design does not rely on any specific property of a particular disease, patient population, or clinical data set. PredicT-ML is generalizable to many data sources by supporting common data models (e.g., OMOP [16]) and their associated standardized terminologies used by many healthcare systems. Once attributes needed to solve a clinical predictive modeling problem are available in one of the common data models or in a structured data set, PredicT-ML can be used to build models. This interoperability enables data integration and helps build machine learning predictive models with big clinical data from multiple healthcare systems.

### Innovation

Existing machine learning tools are not tailored to healthcare researchers’ needs. This work addresses gaps in existing software and is innovative for the following reasons:

1. We present the first software to automate building machine learning predictive models with big clinical data and to support fast iterative machine learning. The software will enable healthcare administrators and researchers to rapidly ask a series of what-if questions when probing opportunities to use predictive models to improve outcomes and reduce costs for various diseases and patient populations. Existing machine learning tools cannot do this.
2. We present a new method to automatically select algorithms, feature selection techniques, and hyper-parameter values for a given machine learning problem more quickly than current methods.
3. We present the first method to automatically and efficiently select temporal aggregation periods and operators for clinical attributes.
4. We present a new method to project in real time model accuracy and estimated patient outcomes of using models as a function of time allotted for model building. This is the first time continuous feedback is provided during automatic model selection.

### Related work

Several dozen methods have been published on automatically selecting machine learning algorithms and/or hyper-parameter values. Few of these methods have been fully implemented and can handle many algorithms and any number of hyper-parameter value combinations. None is efficient for large data sets or optimized for a computer cluster. The automatic machine learning model selection method described in this paper addresses these methods’ limitations. A summary comparison of these methods is provided in our paper [17]. A detailed review of these methods is provided in our paper [8].

Many clinical data are stored in the EAV format [18]. They often must be pivoted into denormalized relational table formats before conducting analysis. We previously developed three techniques for improving efficiency of pivoting [19] and showed how to use MapReduce [20] to implement them on Spark [17]. PredicT-ML uses a similar approach to implement temporal aggregation operators on Spark.

PredicT-ML projects in real time model accuracy and estimated patient outcomes of using models as a function of time allotted for model building. This is a form of progress indicator. Progress indicators are widely used in software systems and have been used before for database SQL queries [21, 22], program compilation [23], and MapReduce jobs [24, 25]. Traditional progress indicators continuously estimate remaining task execution time. In comparison, PredicT-ML continuously projects trend curves.

Haug et al. developed a semi-automated method to systematically compile risk factors for a disease [26], and a tool that first uses compiled knowledge to automatically retrieve data from a clinical data warehouse and organize them without performing temporal aggregation, then uses a machine learning algorithm chosen by the user to build predictive models for the disease [27, 28]. It would be an interesting area for future work to investigate using natural language processing techniques to semi-automatically compile medical knowledge that PredicT-ML needs for automating temporal aggregation.

## Methods

PredicT-ML is built on top of several existing big data software systems, allowing it to run on a single computer or on a computer cluster for parallel processing. Hadoop [29] and Spark [30] are major open source big data software systems supporting the MapReduce framework [20] for distributed computing. MapReduce uses the Map and Reduce functions. The Map function turns an input item into zero or more key-value pairs. The Reduce function turns a key and its list of linked values into zero or more key-value pairs, which can be of another type. Hadoop stores data in the Hadoop distributed file system and typically executes jobs by repeatedly reading and writing data from and to disk, incurring large overhead [31]. To address Hadoop’s limitations and improve performance, Spark [30, 31] was built atop of the Hadoop distributed file system and performs most operations in memory. Spark SQL [32, 33] supports a large part of structured query language (SQL) on top of Spark. MLlib [11, 34, 35] is the distributed machine learning library of Spark. Spark can run machine learning algorithms more than 100 times faster than Hadoop [32]. PredicT-ML is developed using the Spark package and new techniques to overcome existing software’s limitations. The Spark package includes Spark SQL and MLlib.

**Fig. 1:**
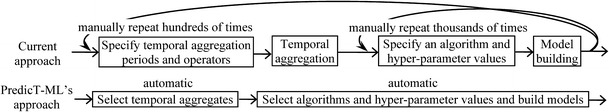
The current approach of building machine learning models vs. PredicT-ML’s

**Fig. 2:**
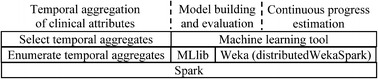
Architecture of PredicT-ML

Figure 1 compares the current approach of building models to PredicT-ML’s. During machine learning on big clinical data, the following steps are executed sequentially: temporally aggregate clinical attributes, select algorithms and hyper-parameter values, build models, and evaluate models. PredicT-ML supports these steps (Fig. 2). The first step is optional, e.g., if the medical data set contains no repeatedly recorded clinical attribute. PredicT-ML is built using the open source software systems Weka and Spark including Spark SQL and MLlib. Each one either is written in Java or supports a Java application programming interface. PredicT-ML is written in Java so it can interface with and call the functions in these software systems.

PredicT-ML integrates machine learning functions of Weka [5] and MLlib [11, 34, 35] by calling the Java application programming interface and/or modifying source code of Weka and MLlib. Weka is a widely used open source machine learning and data mining toolkit including many standard machine learning algorithms and feature selection techniques. distributedWekaSpark [36] is Weka’s distributed computing package for Spark. MLlib implements a subset of the algorithms and feature selection techniques supported by Weka. PredicT-ML supports all algorithms and feature selection techniques in Weka. For an algorithm or technique implemented in MLlib, PredicT-ML uses MLlib’s implementation that integrates with Spark more effectively than distributedWekaSpark’s implementation [36]. For any other algorithm or technique, PredicT-ML uses distributedWekaSpark’s implementation.

PredicT-ML provides an intuitive graphical user interface. In the input interface, the user provides the medical data set’s storage location, such as the name of a file in the Hadoop distributed file system, a comma-separated values (CSV) file in the local file system, or a table in a relational database. PredicT-ML will automatically load the medical data set into Spark before machine learning.

The graphical user interface of Weka [5] supports feature selection (optional) and model building and evaluation. The user specifies in the input interface the data file, independent and dependent variables, and machine learning algorithm and hyper-parameter values. After clicking “Start,” Weka constructs the model and displays its performance metrics. The graphical user interface of PredicT-ML for machine learning works in a similar way with two major differences. First, in the input interface of Weka, the user has to choose an algorithm before model building. Similar to Auto-WEKA [7], PredicT-ML regards the identification of a feature selection technique to be a hyper-parameter and automatically selects the algorithm, feature selection technique, and hyper-parameter values. The user can override PredicT-ML’s choice. Second, to help the user track progress of the automatic model selection process, PredicT-ML displays a curve showing the highest accuracy achieved over time. At any time, the user can stop the process and ask PredicT-ML to output the most accurate model built.

By default, PredicT-ML considers all input variables, feature selection techniques, and machine learning algorithms. The user can choose to specify, in the input interface, a portion of input variables that has to be used in the model. Then feature selection will be employed only to the remaining input variables. Also, the user can choose to specify algorithms and/or feature selection techniques to be examined.

## Results and discussion

This part of the paper presents the detailed design of PredicT-ML and is organized as follows. We first describe three methods used in PredicT-ML (Fig. 2), one to efficiently automate selection of machine learning algorithms and hyper-parameter values, one to efficiently automate selection of temporal aggregation periods and operators for clinical attributes, and one to project in real time model accuracy and estimated patient outcomes of using models as a function of time allotted for model building. Then we describe our preliminary results on the initial implementation and performance evaluation of our draft method for automatically selecting machine learning algorithms and hyper-parameter values [17] on a single computer. Finally, we describe our evaluation plan for PredicT-ML.

### A method to efficiently automate selection of machine learning algorithms and hyper-parameter values

As shown in our review paper [8], few published automatic selection methods for machine learning algorithms and hyper-parameter values [7, 10, 37] have been fully implemented and can handle many algorithms and any number of hyper-parameter value combinations. All of these methods use the Auto-WEKA automatic selection approach [7], but none is efficient for large data sets or optimized for a computer cluster.

To address existing methods’ inefficiency, we recently proposed a draft method to quickly find an effective combination of a machine learning algorithm and hyper-parameter values for a given modeling problem when many algorithms are considered [17, 38]. The method performs progressive sampling [39], filtering, and fine-tuning to quickly reduce the search space. Our idea is to perform fast trials on small samples of the data set to remove as many unpromising combinations as soon as possible, and to allocate more resources to fine-tuning promising ones. We use a limited random sample of the data set to evaluate various combinations and identify several promising ones as the foundation for a reduced search space. A combination is regarded as promising if a model trained using it and the sample achieves accuracy above an initial threshold. We then expand the sample, increase the accuracy threshold, evaluate and fine-tune these combinations on the sample, and identify fewer promising ones as the foundation for a further reduced search space several times. As the sample increases, the search space decreases. In the last round, we use the complete data set to identify an effective combination.

**Fig. 3:**
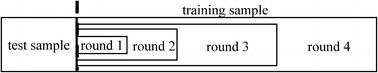
Progressive sampling used in our automatic selection method

More specifically, our draft method proceeds in rounds. Each round uses two disjoint, random samples of the data set: the training sample and test sample. The training sample expands across rounds and doubles its size each round, while the test sample remains the same across rounds (Fig. 3). In the first round, we begin with a small training sample. For every machine learning algorithm, we test both its default hyper-parameter value combination and a fixed number of random ones (e.g., 20), if any. To test a combination, we first use the algorithm and combination to train a model on the training sample, and then estimate the model’s accuracy on the test sample. Across all combinations tested so far for an algorithm, the highest accuracy achieved reflects the algorithm’s potential and is used to identify unpromising algorithms.

In each round after the first, for every remaining machine learning algorithm, we use a Bayesian optimization approach [40] to select multiple new hyper-parameter value combinations, if any, for testing. To guide the search direction, a regression model is constructed to predict machine learning model accuracy based on hyper-parameter values [7, 9, 40, 41]. Removing irrelevant hyper-parameters from the regression model can make it more accurate, reduce tests needed for the search process, and expedite search. For this purpose, we build a separate regression model for each algorithm. Using the expected improvement criterion and a regression model for predicting combination quality, the Bayesian optimization approach selects two groups of new combinations. The first group comes from local search around the 10 previously tested combinations with the largest expected improvement. The second group is the 10 combinations with the largest expected improvement from 10,000 random ones. Combinations in these two groups are interleaved for testing. Combinations in the first group tend to have better quality than those in the second group, as the regression model is better at predicting the quality of combinations close to previously tested ones [42]. To prevent search from being overly delayed on a single combination of an algorithm and hyper-parameter values, we impose a limit *T* on time that can be spent testing a combination. *T* is increased across rounds. Once *T* is exceeded, a test on a combination is stopped.

Our draft method needs improvement to perform satisfactorily and to work well on a computer cluster. For efficiently and automatically selecting machine learning algorithms and hyper-parameter values, we use a new method that is based on the draft method and includes additional techniques for optimization and refinement to further improve efficiency. These techniques have never been used before for automating machine learning model selection.

*Technique 1: Remove unpromising features early*. Both existing automatic selection methods [7, 10, 37] and our draft method conduct many tests. Each test checks a feature selection technique, machine learning algorithm, and hyper-parameter value combination on (a subset of) the data set. Since execution time of feature selection and model building grows at least linearly with the number of features, conducting the test is slow if the data set has many features. In this case, most features likely have low predictive power. Before conducting tests, we apply a fast feature selection technique such as the information gain method to (a sizeable sample of) the complete data set and quickly remove features unlikely to be predictive. Then each test can use a slower feature selection technique for a finer examination of the remaining features.

*Technique 2: Drop unpromising feature selection techniques early*. Our draft method builds a separate regression model for each machine learning algorithm to predict machine learning model accuracy based on hyper-parameter values. Separation helps make the regression model more accurate, but each regression model uses all hyper-parameters for feature selection, which are too many and reduce its accuracy.

Each feature selection technique uses a feature search method and feature evaluator [7]. The hyper-parameters for feature selection include the choice of feature search method, the choice of feature evaluator, and hyper-parameters for each feature search method and evaluator. To further reduce hyper-parameters used in each regression model, we conduct fast trials on small samples of the data set to remove as many unpromising feature evaluators as early as possible. Once a feature evaluator is removed, its hyper-parameters are dropped from all regression models thereafter. Also, it will not be used in the rest of the search process. This reduces the search space and tests needed for the search process. We remove feature evaluators over rounds until no more than a fixed number of feature evaluators (e.g., 4) remain. Feature search methods can be handled similarly. This technique can be applied to several other parts of a data analytic pipeline [43] such as handling missing values and imbalanced classes.

We identify and remove unpromising feature evaluators similarly to our draft method identifies and removes unpromising machine learning algorithms. Our draft method proceeds in rounds. In each round, across all hyper-parameter value combinations tested so far for an algorithm, the highest accuracy achieved reflects the algorithm’s potential and is used to identify unpromising algorithms. Since the choice of feature evaluator is a hyper-parameter, we draw the following analogy. Across all combinations of algorithms and hyper-parameter values tested so far for a feature evaluator, the highest accuracy achieved reflects the feature evaluator’s potential. However, unlike the case with algorithms, our draft method makes no guarantee about the number of tests done on each feature evaluator. Without sufficient tests, our estimate of a feature evaluator’s potential can be highly inaccurate. To address this issue, we modify our draft method. If the number of tests done on a feature evaluator is smaller than a fixed number (e.g., 20), we perform additional tests for the feature evaluator to make up the difference. Each additional test uses a random combination of an algorithm and hyper-parameter values and is fast to perform, as the training sample is small in the first round.

*Technique 3: Improve the quality of combinations selected from random ones*. As mentioned above, in each round after the first, for every remaining machine learning algorithm, our draft method selects two groups of new hyper-parameter value combinations for testing. Combinations in the first group tend to have better quality than those in the second group. If we can improve combination quality in the second group, we can improve the final search result’s quality. Or, we can reduce tests for later rounds and improve search speed.

Our draft method doubles the training sample size each round. To improve the quality of hyper-parameter value combinations in the second group, we conduct fast trials on small samples of the data set to identify unpromising combinations. Starting from the third round when the training sample is no longer small, we select the 30 combinations with the largest expected improvement from 10,000 random ones. Each of the 30 combinations is tested on the training sample from the first rather than the current round. Each test is fast to perform, as the training sample from the first round is small. Among the 30 combinations, the 10 achieving the highest accuracies tend to have better quality than the other 20 and form the second group. For each of the other 20 combinations, we use the scaling technique in our paper [17] to perform scaling for each round and obtain a rough accuracy estimate for the combination for the current round. In this way, without wasting any test results, all 30 combinations are used for building the regression model in later rounds.

*Technique 4: Automatically build an ensemble model from models constructed during the search process*. The search process produces many machine learning models. Once complete, we use the approach in Caruana et al. [44] to automatically build an ensemble model from these models with low overhead. The ensemble model is often more accurate than individual models and can serve as the final model. Feurer et al. [37] used a similar method to form an ensemble model after the search process, but limited the search process to base models, affecting accuracy of models produced during it. In contrast, we use a novel approach that has no such restriction and produces an ensemble model likely to be more accurate. Almost all of the models produced during our search process are trained using parts of the data set, while model accuracy generally improves with a larger training set. To further improve our ensemble model’s accuracy, we can use the whole data set to train a few of the most accurate models to make them even more accurate.

*Technique 5: Parallelize the tests in the first few rounds of the search process*. Our draft method performs tests sequentially. To speed up the search process, we can parallelize the tests in the first few rounds, and then switch to parallelize data processing for each test one by one in later rounds. Our discussion focuses on the case that PredicT-ML runs on a cluster of computers. The case that PredicT-ML runs on a computer with multiple processing units (e.g., processors or processor cores) can be handled similarly.

Our draft method proceeds in rounds. Except for the final one, each round tests multiple combinations of machine learning algorithms and hyper-parameter values one by one. The training sample expands across rounds. In each of the first few rounds, the training sample is small. Partitioning it across all computers and using each combination to train a model on all computers concurrently will incur excessive communication overhead. To use parallel processing well, we test different combinations on different computers concurrently.

We choose a positive integer *m* (e.g., 3) < the total number of rounds. *m*’s value increases with the search space size partly reflected by the number of machine learning algorithms. Before starting, we distribute a copy of both the training sample for the *m*-th round and the test sample to each computer. The training sample for the *m*-th round is a super set of that for each earlier round. Thus, in each of the first *m* rounds, a copy of both the training sample for the current round and the test sample is available on each computer. All combinations of algorithms and hyper-parameter values to be tested are put into a queue [45]. When a computer becomes available, the first combination is removed from the queue and sent to the computer for testing. In this way, all combinations are partitioned across all computers roughly evenly for concurrent testing.

Starting from the (*m* + 1)-th round, the training sample becomes large. Communication overhead is no longer excessive. Also, the number of combinations of machine learning algorithms and hyper-parameter values to be tested in each round becomes small. In this case, we can no longer use the power of parallel processing well by testing different combinations on different computers concurrently. For example, some computers will stay idle if a specific round has fewer combinations to test than computers. Before starting the (*m* + 1)-th round, we partition the whole data set evenly across all computers, if not done before. Starting from the (*m* + 1)-th round, the training sample is already partitioned across all computers. We test combinations one by one and use each combination to train a model on all computers concurrently.

If the cluster has many computers, in the (*m* + 1)-th round the training sample is no longer small, but partitioning it across all computers will still incur excessive communication overhead in model training. To use parallel processing well, we add one or more intermediate transition stages between rounds. In each round of such a stage, all computers are partitioned into multiple subsets. Different combinations of machine learning algorithms and hyper-parameter values are tested on different subsets. For each combination, we train a model on all computers in its corresponding subset concurrently.

*Technique 6: Skip overly time*-*consuming tests*. Our draft method imposes a limit *T* on time that can be spent testing a combination of a machine learning algorithm and hyper-parameter values. Once *T* is exceeded, a test on a combination is stopped, often with no accuracy recorded and time spent on the test wasted. In practice, multiple good combinations achieving similar accuracies often exist. Some can be tested much more quickly than others, and finding any of them would serve our purpose. To avoid wasting time on overly time-consuming tests, we predict the time needed for a test and skip tests unlikely to finish within *T*. We use a fixed factor *f* > 1 to tolerate some imprecision in prediction. If the predicted test time for a combination is > *f* × *T*, we regard the combination as unlikely to finish within *T* and skip it. By spending the time testing other combinations likely to finish within *T*, we can obtain accuracy results for more combinations within the same total time. This can improve the final search result’s quality or expedite our search process.

We use the following method to predict the test time for a combination of a machine learning algorithm and hyper-parameter values. Our discussion focuses on model training time. Feature selection time can be handled similarly. For each algorithm, its default hyper-parameter value combination is tested in the first round. The test time serves as the baseline. We model the ratio of the amount of time for each other test to the baseline as the product of two ratios, one for training sample size and another for the algorithm’s hyper-parameter values. The formula describing the relationship between training sample size and model training time is known for many algorithms and used to compute the ratio for training sample size. For each other algorithm, we use results from previous machine learning problems to build a regression model for that ratio.

For estimating the ratio for a machine learning algorithm’s hyper-parameter values, we compare two approaches and pick the more accurate one for each algorithm. The first approach is to build a random forest model that can deal with both continuous and categorical hyper-parameters. In comparison, Snoek et al. [9] used a Gaussian process to predict the test time for a hyper-parameter value combination. That approach cannot handle categorical hyper-parameters or a training sample that expands across rounds. The second approach is to compute this ratio as the product of multiple ratios, one for each hyper-parameter’s value. For a hyper-parameter known to impact model training time little, we set the ratio for its value at one. For a hyper-parameter with a known formula describing the relationship between its value and model training time, we use the formula to compute the ratio for its value. For each other hyper-parameter, we use results from previous machine learning problems to fit a (e.g., power) function for the ratio for its value. Or, we can specifically treat the categorical hyper-parameters known to greatly impact model training time. For each combination of their values, we make a separate fit of functions for the ratios for the other hyper-parameters’ values.

*Technique 7: Delay time*-*consuming tests to the end of the search process*. Certain hyper-parameters of some machine learning algorithms are known to have a special property. Setting the hyper-parameter to a value in a specific range, often in a specific direction, is likely to improve model accuracy at the expense of much slower model training. An example of such hyper-parameters is the number of decision trees in a random forest. A random forest is an ensemble of decision trees. Using many decision trees often improves model accuracy, but model training time increases proportionally with the number of decision trees. To ensure the search process can test many combinations of algorithms and hyper-parameter values within reasonable time, we cannot afford testing hyper-parameter values in such ranges during the search process. However, we can do such tests at the end of the search process when model training time is no longer a major issue. For the top few algorithms achieving the highest accuracies and with the special property, we start another search process based on the most accurate models built using them, test their hyper-parameter values in such ranges, and try to use them to build even more accurate models. This can improve the final model’s accuracy without affecting accuracies of the models built during the first search process. In the second search process, if we know that changing a numerical hyper-parameter’s value in a specific direction is likely to improve model accuracy, we can move in that direction until we have confidence in convergence [39]. That is, model accuracy no longer improves (much) as the hyper-parameter’s value changes in that direction.

### A method to efficiently automate selection of temporal aggregation periods and operators for clinical attributes

Manually specifying temporal aggregation periods and operators is labor-intensive and time-consuming. Our idea for automating temporal aggregation on a clinical attribute is to list temporal aggregation periods and operators that can be clinically meaningful, enumerate all aggregation period and operator pairs, then conduct feature selection to select one or more most predictive pairs. Our automation method requires disease-specific knowledge compiled by clinical experts and stored in PredicT-ML. Knowledge can be added over time and does not have to be comprehensive or precise for PredicT-ML to be useful. PredicT-ML can function once some aggregation operators are compiled. For selecting aggregation periods, the shortest and longest possible ones serving as search ranges can be specified conservatively. A crowd sourcing approach can be adopted for compiling knowledge.

We demonstrate our techniques by compiling knowledge for asthma and type 2 diabetes. We start from commonly used aggregation operators and clinical attributes related to these two diseases. In the future, we plan to (1) compile additional knowledge for these two diseases to cover more aggregation operators and clinical attributes, (2) compile knowledge for other diseases so PredicT-ML can automate temporal aggregation for them, and (3) add an interface allowing users to input knowledge into PredicT-ML. PredicT-ML can automate temporal aggregation for a new disease and prediction target pair once knowledge is input for it.

#### Compile knowledge

By combining our medical knowledge, our extensive experience building clinical predictive models, literature on clinical predictive modeling [46, 47] and temporal pattern mining [48, 49], and experience of colleagues at the University of Utah, we compile a list of commonly used temporal aggregation operators such as in the aggregation period: (1) the clinical attribute’s minimum/maximum/average value, (2) whether the clinical attribute (e.g., a disease diagnosis code) appears, (3) the duration of using the clinical attribute (e.g., a medication), (4) the count of the clinical attribute (e.g., hospitalization), (5) whether the clinical attribute’s value (e.g., weight) is monotonically increasing/decreasing, (6) the clinical attribute’s most recent value, (7) the (relative) change in the clinical attribute’s value, (8) the percentage of the clinical attribute’s values (e.g., lab test results) that are high/low/normal/equal to a given value, (9) whether the quarterly/monthly/weekly frequency of having the clinical event (e.g., doctor visits) is monotonically increasing/decreasing, and (10) whether a medication’s use is intensified/de-intensified. We implement all of the operators on Spark that currently do not exist.

Suitable temporal aggregation periods and operators vary by diseases, prediction targets, and clinical attributes. Many combinations of diseases, prediction targets, and clinical attributes exist. Our automation method needs knowledge for each combination. Given limited resources, we use a grouping technique to reduce efforts needed for compiling knowledge. Instead of compiling knowledge for each combination, we partition combinations into groups and compile knowledge for each group.

Consider the targeted diseases for which we want PredicT-ML to automate temporal aggregation. We check the clinical predictive modeling literature and find commonly used prediction targets for targeted diseases, as we did for bronchiolitis [50]. The clinical experts in our research team use their expertise and reference grouper models such as the Clinical Classifications Software (CCS) system [51, Chapter 5] to group prediction targets with similar properties and repeatedly recorded clinical attributes with similar properties. For each potentially related combination of a disease, prediction target group, and clinical attribute group, our clinical experts select suitable temporal aggregation operators from our list, partition them into categories, and pick the shortest and longest, clinically meaningful temporal aggregation period and a progression method for each category. The progression method specifies how to automatically generate a sequence of candidate aggregation periods. In an exponential progression method, we start from the shortest aggregation period and elongate the period’s length exponentially (e.g., by doubling) until we reach the limit allowed by the longest aggregation period. In an arithmetic progression method, in each step we elongate the period’s length by a constant.

Temporal aggregate values are used as features to build predictive models, where features containing non-redundant information are generally preferred [52]. To facilitate this, we partition temporal aggregation operators into categories. The operators in the same and different categories produce relatively redundant and non-redundant aggregate values, respectively. For a clinical attribute, our automation method produces an aggregate value from each category of operators rather than from each individual operator. The aggregate values from different categories provide non-redundant information that can help increase model accuracy. For instance, for a patient’s weight, two non-redundant aggregate values are (1) the average weight and (2) whether the patient’s weight is monotonically increasing in the aggregation period. Two relatively redundant aggregate values are the change and relative change in a patient’s weight in the aggregation period.

Many combinations of a disease, prediction target group, and clinical attribute group exist. It is non-trivial to compile knowledge for each combination. However, knowledge overlaps across different combinations. For example, for the same clinical attribute group, aggregation periods and operators overlap for different diseases. Compilation time for each new combination will decrease as compiled knowledge is applied to new combinations.

#### Select temporal aggregation periods and operators

Given a disease, prediction target, and medical data set, we use a feature selection technique to automate temporal aggregation on clinical attributes. We identify the prediction target group including the prediction target. For each clinical attribute in both the medical data set and a clinical attribute group related to the disease and prediction target group, we automatically select one temporal aggregation operator from each related operator category, and a temporal aggregation period between the shortest and longest ones linking to the category.

To do this, we generate a sequence of temporal aggregation periods between the shortest and longest periods based on the progression method linking to the aggregation operator category. We list all operators in the category and all aggregation period and operator pairs. On a large sample of the whole data set, we use each pair to compute an aggregate value feature of the clinical attribute, then apply a feature selection technique [52] such as the information gain method to select the feature most predictive of the prediction target. The pair linking to this feature is used to compute this feature on the whole data set. Finally, we use Spark SQL [32, 33] to join the selected aggregate value features with non-repeatedly recorded clinical attributes. Basically, our method selects one feature from each feature group. This is different from the traditional methods of selecting features with group structures [52], where multiple features in the same group tend to be selected or not selected together with no limit imposed on the number of features selected from any single group. The selected aggregate value features are stored in the Hadoop distributed file system, Spark’s default persistent storage space. In PredicT-ML’s input interface, the user can modify their storage location, e.g., to export them to CSV format for use by other programs.

**Fig. 4:**
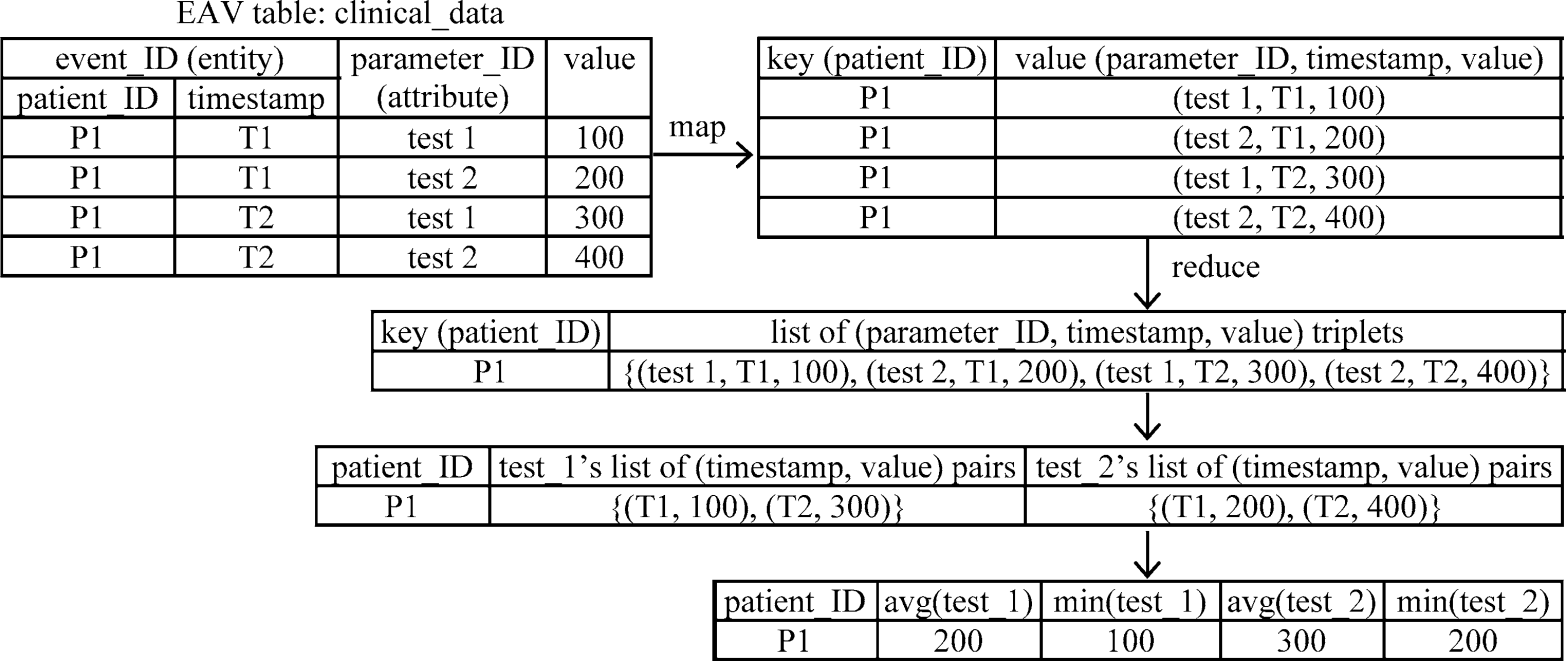
Use MapReduce to obtain temporal aggregate values of two clinical parameters ‘*test 1*’ and ‘*test 2*’

Repeatedly recorded clinical parameters are often stored in the EAV (Entity-Attribute-Value) format [17, 18], which uses tables including at least three parts: the entity, attribute, and value (Fig. 4). Typically, the entity combines patient ID and timestamp and identifies a clinical event [14, p. 58]. The attribute identifies a clinical parameter. The value includes the clinical parameter’s value. As a result, an EAV table merges numerous clinical parameters and their values in the attribute and value parts. We use MapReduce [20] to compute all temporal aggregate value features together on Spark. The Map function forms one key-value pair per EAV tuple. The key is the patient ID in the EAV tuple. The value combines the attribute, timestamp, and value in the EAV tuple. In the Map function, EAV tuples corresponding to unused clinical parameters are removed early on [19]. For a patient ID, the Reduce function merges all relevant EAV tuples with the patient ID from one or more EAV tables, collects all (timestamp, value) pairs for each clinical parameter, computes each aggregate value feature of every clinical parameter, and combines all aggregate value features into a relational tuple.

Different medical data sets use different schemas, medical terminologies, and medical coding systems, creating difficulty in using pre-compiled knowledge. To address this issue, PredicT-ML’s automated temporal aggregation function requires the medical data set excluding the prediction target to conform to the OMOP common data model [16] and its associated standardized terminologies [53]. As this data model standardizes administrative and clinical attributes from at least 10 large healthcare systems in the U.S. [54], PredicT-ML can be used on medical data sets from these systems. In the future, we plan to add support for the i2b2 [55] and PCORnet common data models [56] into PredicT-ML.

Our above discussion focuses on the case that each aggregation operator acts on one clinical attribute without using any other parameter beyond the aggregation period. The case that an aggregation operator acts on >1 clinical attribute and/or uses other parameters beyond the aggregation period can be handled similarly, e.g., by enumerating values for the other parameters. An example of such aggregation operators is whether a given medication (e.g., fluconazole) was prescribed in a certain period before an abnormal result of a given (e.g., liver function) lab test occurred, reflecting the knowledge of this side effect. Here, the medication and lab test result are two clinical attributes. The threshold for the time gap between the medication’s prescription and the abnormal lab test result’s occurrence is a parameter beyond the aggregation period. Two other examples of such aggregation operators are whether medications are switched and whether multiple therapies are used.

Also, our above discussion focuses on the case that the medical data set includes exactly one prediction target instance per patient. The case that on some or all patients, the medical data set includes more than one prediction target instance per patient can be handled similarly. There, each prediction target instance of a patient corresponds to a different time point, for which a separate set of temporal aggregate values will be computed. For example, suppose we have 5 years’ data on each patient. Our goal is to use the previous 3 years’ data to predict acute care use within the next year. For predicting acute care use within year 4, data in years 1–3 are used to compute temporal aggregate values. For predicting acute care use within year 5, data in years 2–4 are used to compute temporal aggregate values.

### A method to project in real time model accuracy and estimated outcomes of using models as a function of time allocated for model building

**Fig. 5:**
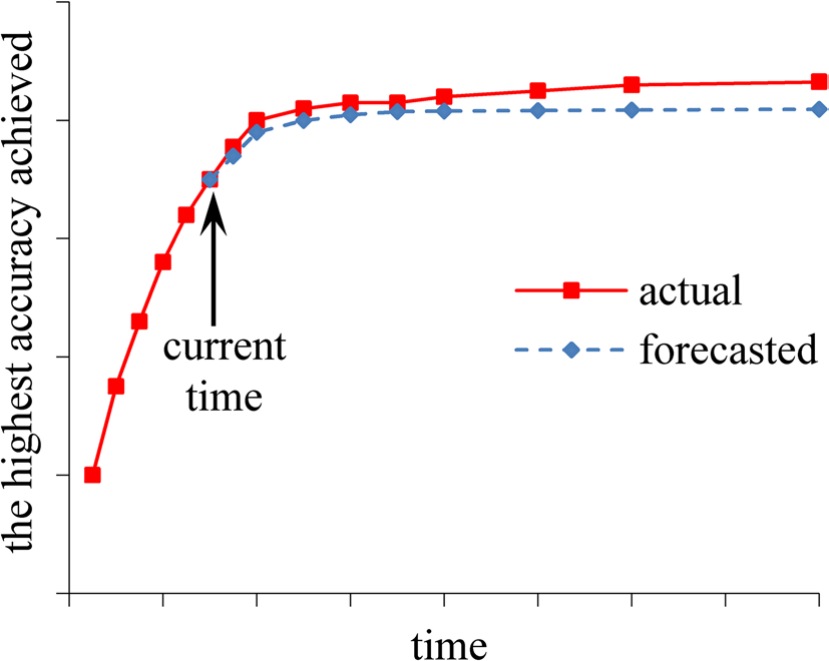
The highest model accuracy achieved by PredicT-ML over time

To be more user-friendly and useful, during the automatic model selection process, PredicT-ML displays projections of model accuracy and estimated patient outcomes of using models as a function of time allotted for model building (Fig. 5). At any time among all models built thus far, the one obtaining the best accuracy is used to obtain model accuracy and estimated outcomes of using the best model for the current point in time. As the process proceeds, the projections are continuously refined to become more precise.

The highest model accuracy achieved by PredicT-ML increases over time, whereas the rate of increase typically decreases over time (Fig. 5). To model the highest model accuracy achieved over time, we use an inverse power law function of the form f(*t*) = *a* − *b* × *t*^−*c*^ [57]. Here, *t* denotes time, 0 < *b* < *a* < 1, and *c* > 0. The inverse power law function has been used before to model the relationship between model accuracy and training set size [57]. In our case, the function is re-fitted from time to time to project model accuracy vs. time allotted for model building.

More specifically, each time PredicT-ML builds a predictive model during the automatic model selection process, we obtain a training data point of time and the highest model accuracy achieved. We use the training data points obtained so far to fit the inverse power law function in a way similar to that in Figueroa et al. [57]. Our goal of fitting the function is to predict the future as precisely as possible. Our goal is not to minimize the average distance between all training data points obtained so far, which represent the past, and the fitted curve. In general, the training data points obtained later reflect the trend into the future better than those obtained earlier. Based on this insight, we give larger weights to the training data points obtained later rather than treating all training data points equally. We fix a positive integer *g* (e.g., 20). Let *h* represent the number of training data points obtained so far. When fitting the function, we consider only the last *n* = min(*g*, *h*) training data points and give a weight of *i*/*n* to the *i*-th of the *n* data points [57].

For a future time point, we use a method similar to the one in our papers [1, 58] to obtain projections of estimated outcomes of using predictive models. Our discussion focuses on a categorical prediction target. Continuous prediction targets can be handled similarly through discretization. We compute the percentage of instances *q*_*c*_ in each category *c* of the prediction target. For each category pair (*c*_*1*_, *c*_*2*_), the user of PredicT-ML specifies the outcome $o_{{c_{1} , c_{2} }}$ (e.g., cost) when an average instance (e.g., patient) in *c*_*1*_ is predicted to be in *c*_*2*_ and interventions are decided based on the prediction result. We use a function fitting approach similar to the one above to project $p_{{c_{1} , c_{2} }}$, the percentage of instances in *c*_*1*_ that the best model at this time point will predict to be in *c*_*2*_. When *c*_*1*_ = *c*_*2*_, $p_{{c_{1} , c_{2} }}$ reflects accuracy and tends to increase over time. Thus, a function of the form f(*t*) = *a* − *b* × *t*^−*c*^ is used. When *c*_*1*_ ≠ * c*_*2*_, $p_{{c_{1} , c_{2} }}$ reflects error rate and tends to decrease over time. Thus, a function of the form f(*t*) = *a* + *b* × *t*^−*c*^ is used, where *b* is >0 but not necessarily < *a*. The estimated outcome of using the best model at this time point is projected to be $\sum_{{c_{1} , c_{2} }} q_{{c_{1} }} \cdot p_{{c_{1} , c_{2} }} \cdot o_{{c_{1} , c_{2} }}$, where $q_{{c_{1} }} \cdot p_{{c_{1} , c_{2} }}$ is the expected percentage of instances that are in *c*_*1*_ and will be predicted to be in *c*_*2*_.

#### Preliminary results

Recently, we did an initial implementation and performance evaluation of our draft method for automatically selecting machine learning algorithms and hyper-parameter values [17] on a single computer. Compared to the state of the art Auto-WEKA automatic selection method [7], on 21 prominent machine learning benchmark data sets, on average our draft method reduced (1) search time by ninefold, (2) classification error rate by 14 % and (3) standard deviation of error rate due to randomization by 26 %. A detailed report of our initial implementation and preliminary results is available in our paper [38]. At present, we are still in the process of tuning our draft method. PredicT-ML’s automatic machine learning model selection function is based on our draft method. Thus, we will not be able to start building PredicT-ML until we have finished tuning our draft method, integrated it with MLlib and distributedWekaSpark’s machine learning functions, and implemented it on top of Spark on a computer cluster, which will take quite some time.

#### Evaluation plan

PredicT-ML is a large software system that will take several years to be fully built. This section presents our evaluation plan for PredicT-ML. We will evaluate each of the three methods described above.

#### Demonstration test cases

For evaluating each of the three methods, we will use the same three test cases, where PredicT-ML will be used to build models to predict asthma outcomes and risk for type 2 diabetes. PredicT-ML can be used for other diseases and prediction targets. We choose these test cases based on the following criteria: the data sets are readily accessible, the addressed diseases are common, both categorical and continuous prediction targets are covered, and resulting predictive models can potentially be deployed to impact care delivery. Asthma and type 2 diabetes each have a prevalence of 9 % [59–62] and account for significant healthcare use and cost [1, 4]. Accurate prediction of asthma outcomes and risk for type 2 diabetes helps target preventive interventions to appropriate patients to improve health outcomes and reduce costs [1, 4]. For example, each year asthma incurs 439,000 hospitalizations, 1.8 million emergency department visits, 3630 deaths [61], and 56 billion dollars in cost [63]. Case management for the right asthma patients can lower cost by up to 15 % and reduce emergency department visits and hospital (re)admissions by up to 30–40 % [64–70].

*Computing environment* All experiments will be performed on the HIPAA-compliant Homer computer cluster at the University of Utah [71]. With appropriate authorization, all members in our research team at the University of Utah can access this cluster using university computers. Our analysis results will form a base to expand testing of PredicT-ML on other clinical data sets and test cases in the future.

##### Test case 1: predict acute care use in asthmatic children within the next year

*Patient population* Intermountain Healthcare patients in the past 11 years with asthma, identified by the method in Schatz et al. [72–74] as having (1) at least one ICD-9 diagnosis code of asthma (493.xx) or (2) ≥ 2 “asthma-related medication dispensings (excluding oral steroids) in a one-year period,” “including β-agonists (excluding oral terbutaline), inhaled steroids, other inhaled anti-inflammatory drugs, and oral leukotriene modifiers” [72]. Intermountain Healthcare has 22 hospitals and 185 clinics and is the biggest healthcare system in Utah.

*Data set* The Intermountain Healthcare enterprise data warehouse contains a large set of attributes and ~9000 tables [75]. On an encrypted and password-protected computer cluster, we will conduct secondary analysis of a large, de-identified, administrative and clinical data set obtained from the Intermountain Healthcare enterprise data warehouse. The data set includes information about patient encounters in the previous 11 years. For the most recent 5 years, data captured cover ~27,000 children (age 0–17) and ~75,000 adults (age ≥18) with asthma per year. The data set has ~400 attributes partly listed in our paper [1] and is the electronic record of about 85 % of pediatric care and about 60 % of adult care provided in Utah [76, 77]. Identifying asthma requires prescription and refill information. Our data set has this information because Intermountain Healthcare uses its own health insurance plan called SelectHealth. If too many refill records are missing from the Intermountain Healthcare enterprise data warehouse, we will compensate using claim data from the all-payer claims database [78].

*Information about the predictive models* Administrative and clinical attributes will be used to construct models. The prediction target (i.e., dependent variable) is whether acute care (emergency department visit, urgent care, and inpatient stay) with a primary diagnosis of asthma (ICD-9 code: 493.xx) happened to an asthmatic child in the following year [1, 72, 79]. We will use the first 10 years’ data to build models and the 11-th year’s data for testing to obtain the final accuracy estimate of a model, reflecting use in practice. The standard area under the receiver operating characteristic curve (AUC) [5] performance metric will be used. We will adopt standard techniques, e.g., imputation to deal with missing values and identify and remove/correct invalid values [2, 80]. We will adopt grouper models such as the CCS system to group drugs, procedures, and diseases and reduce features [51, Chapter 5].

##### Test case 2: predict individual patient cost in asthmatic adults within the next year

Test case 2 differs from test case 1 in the following ways. The prediction target is an asthmatic adult’s total cost in the following year [51, 81]. Each medical claim links to a billed cost, an Intermountain-internal cost, and a reimbursed cost [51, p. 43]. We will use the Intermountain-internal cost [77], which is less subject to variation resulting from member cost-sharing [51, p. 45] and more closely reflects actual cost. To address inflation, we will use the medical consumer price index [82] to standardize all costs to 2015 dollars. The standard *R*^*2*^ performance metric will be used [81].

##### Test case 3: predict type 2 diabetes diagnosis in adults within the next year

*Patient population and data set* We have used the clinical and administrative data set from the Practice Fusion diabetes classification competition [13]. The data set is publicly available and de-identified. It includes both historical 3-year records (2009–2012) and the next year’s labels of 9948 adult patients in all 50 states in the United States. In the next year, 1904 of these patients were diagnosed with type 2 diabetes. The data set contains information on patient demographics, allergies, immunizations, diagnoses, lab results, smoking status, medications, and vital signs.

*Information about the predictive models* The prediction target is whether a patient had type 2 diabetes diagnosis in the following year. We will randomly choose 2/3 of patients as the training set to build models and use the other 1/3 as the test set to evaluate a model’s performance. Other details mirror test case 1.

#### Performance evaluation of our method for automatically selecting machine learning algorithms and hyper-parameter values

We will implement in PredicT-ML and then compare our method and the state of the art Auto-WEKA method for automatically selecting machine learning algorithms and hyper-parameter values [7]. We will perform temporal aggregation of clinical attributes manually for both methods, as the Auto-WEKA method cannot automate temporal aggregation. We will allow the Auto-WEKA method to test 1000 combinations of algorithms and hyper-parameter values, which according to our experience is essential to find an effective combination. We will test the hypothesis that our method will produce models with equal or higher accuracy (AUC or *R*^*2*^) in ≤1/20 of the time taken by the Auto-WEKA method. We will regard this part of the study partly successful if the hypothesis is confirmed for two test cases, and completely successful if confirmed for all three test cases.

For test case 1, consider the categorical prediction target of acute care usage with two values (classes). A model for this prediction target usually achieves an AUC far below 0.8 [72, 83]. Using a one-sided Z-test at a significance level of 0.05 and assuming for both classes a correlation coefficient of 0.6 between the prediction results produced by our method and the Auto-WEKA method, a sample size of 1305 instances per class has 90 % power to conclude that with an equivalence margin of 3 %, our method produces no worse AUC than the Auto-WEKA method. The 11th year’s data include about 27,000 children with asthma, providing adequate power to test our hypothesis. The cases with test cases 2 and 3 are similar.

#### Performance evaluation and sample size justification for our automated temporal aggregation method

We will test the hypothesis that our automated temporal aggregation method will produce more accurate models than a clinical expert. A clinical and medical informatics expert in our research team will convert all medical data sets into the OMOP common data model format and its associated standardized terminologies. For each test case, we will use PredicT-ML to automatically select machine learning algorithms and hyper-parameter values and compare the accuracies achieved by two predictive models. The first model will be built using our automated temporal aggregation method. The second model will be built with this expert in our research team specifying temporal aggregation periods and operators for up to 20 trials. To avoid potential evaluation bias, this expert will not participate in compiling knowledge for PredicT-ML. We will accept the hypothesis if the first model attains higher accuracy (AUC or *R*^*2*^) than the second model by ≥5 %. We will regard this part of the study partly successful if the hypothesis is confirmed for two test cases, and completely successful if confirmed for all three test cases.

For test case 1, consider the categorical prediction target of acute care usage that has two values (classes). A model for this prediction target typically achieves an AUC well below 0.8 [72, 83]. Assuming for both classes a correlation coefficient of 0.6 between the prediction results of the two models and using a two-sided Z-test with a significance level of 0.05, a sample size of 561 instances per class has 90 % power to identify a difference of 0.05 in AUC between the two models. The 11th year’s data incorporate about 27,000 children with asthma, giving adequate power to test our hypothesis. The cases with test cases 2 and 3 are similar.

#### Performance evaluation of our method for projecting model accuracy

For the same prediction target, the outcome can differ by the specific healthcare application. Hence, our testing focuses on model accuracy rather than estimated outcomes of using models. In projecting model accuracy vs. training set size, the inverse power law model typically produces a root mean squared error <2 % [57]. The highest model accuracy achieved by PredicT-ML vs. time follows a similar trend as model accuracy vs. training set size. Hence, we would expect the inverse power law model to produce reasonable projection accuracy in our case. Our goal is that after ≤1 h, our projected model accuracy (AUC or *R*^*2*^) for the next 3 days or until the automatic model selection process finishes, whichever is first, will differ from the actual accuracy by ≤3 % in root mean squared error [57]. We will regard this part of the study partly successful if the goal is reached for two test cases, and completely successful if reached for all three test cases.

### Ethics approval

We have already obtained institutional review board approvals from Intermountain Healthcare and the University of Utah for evaluating PredicT-ML.

## Conclusions

We describe the design of PredicT-ML, a software system that automates building machine learning predictive models with big clinical data, with continuous feedback to users to help them decide whether they should continue the automatic model building process or consider other modeling options. PredicT-ML can (1) efficiently automate selection of machine learning algorithms and hyper-parameter values, (2) efficiently automate selection of temporal aggregation periods and operators for clinical attributes, and (3) in real time, display projected model accuracy and estimated patient outcomes of using models as a function of time allotted for model building. PredicT-ML will open the use of big clinical data to thousands of healthcare administrators and researchers, and boost the ability to advance clinical research and improve healthcare. We are currently in the process of writing a detailed design document of PredicT-ML.

## Abbreviations

AUC: area under the receiver operating characteristic curve
CCS: clinical classifications software
CSV: comma-separated values
EAV: entity–attribute–value
PredicT-ML: prediction tool using machine learning
SQL: structured query language
